# Assessing the Reliability of a Novel Eye Tracking Test to Measure Fatigue in Athletes

**DOI:** 10.3390/sports13030071

**Published:** 2025-03-03

**Authors:** Anthea Clarke, Clare MacMahon, Todd Pickering, Matthew Driller

**Affiliations:** 1 Sport, Performance, and Nutrition Research Group, School of Allied Health, Human Services, and Sport, La Trobe University, Melbourne 3086, Australia; a.clarke@latrobe.edu.au (A.C.); c.macmahon@latrobe.edu.au (C.M.); m.driller@latrobe.edu.au (M.D.); 2 Department of Psychology, Counselling and Therapy, School of Psychology and Public Health, La Trobe University, Melbourne 3086, Australia

**Keywords:** smooth pursuit test, oculomotor function, EyeGuide Focus, cognitive testing, athlete monitoring

## Abstract

**Background/Objectives:** The study had two objectives: first, to assess the intra- and inter-day reliability of a novel eye tracking device (EyeGuide Focus) in healthy adults; and second, to explore its applicability in measuring fatigue associated with physical strain, pre- and post-rugby match. These objectives were investigated in a two-part study. **Methods:** For Part A, 20 healthy participants (*M*_age_ ± SD = 30 ± 7 years) completed morning and afternoon testing with the EyeGuide Focus over two consecutive days (Day 1 and Day 2) and one day a week later (Day 8). For Part B, 12 female participants (*M*_age_ ± SD *=* 25 ± 5 years) completed EyeGuide Focus measurements pre- and post-rugby union match. **Results:** The results indicate moderate-to-high intra-day and inter-day reliability (ICCs 0.58–0.79). Fatigue induced by a rugby union match did not significantly alter EyeGuide Focus scores (*p* > 0.05), suggesting stability in measurements despite physical exertion. **Conclusions:** The sensitivity of the EyeGuide Focus to minor variations in fatigue warrants further investigation as a tool to aid monitoring and performance.

## 1. Introduction

Oculomotor function appears to be fully developed by adolescence [1] before starting to decline in older age [2]. Major disruptions to oculomotor function can occur due to diseases, such as multiple sclerosis and diabetes, or from traumatic brain injury, such as concussions [3]. Minor and reversible disruptions to oculomotor function can also result from central fatigue due to exercise, stress, or lack of sleep [1,4,5]. These disruptions to oculomotor function can be measured using various tests and observations; however, the most common test for oculomotor function used within a concussion setting is a visual tracking task either in a circular trajectory or in horizontal/vertical directions [6].

Smooth pursuit eye tracking tests (either in circular or horizontal/vertical directions) for pitch-side concussion testing have previously included crude methods, such as the ‘follow my finger’ test with visual observation of the patients’ eye movement. However, with the development of technology, several computer- and tablet-based programs are now available that can objectively track pupil movement in response to a guide on the screen, with a quantified value for the deviation from the expected tracking. The EyeGuide Focus (https://www.eye.guide/eyeguide-focus, accessed on 20 February 2025) is one such device that uses smooth pursuit eye tracking displayed on an iPad, with pupil movement tracked by a camera positioned close to the face. This device is portable, simple to set up, and keeps everything needed to run it together in one case, allowing for easy and quick assessment of players wherever they are. In a small sample of concussed individuals, the EyeGuide Focus showed results following a concussion were over two times higher than healthy baseline scores of a wider population sample [7]. More recent work using the device, however, is more conflicting, with Pearce et al. [8] showing worse scores on the EyeGuide Focus 48 h post-concussion compared to baseline in an ice hockey and Australian football sample, while Fuller et al. [9] showed no difference in EyeGuide Focus scores between concussed and non-concussed rugby players. Outside of concussion, the EyeGuide Focus also shows substantially higher scores as a result of a 24 h shift in surgeons compared to their baseline and at 12 h [10]. These results suggest this device may be useful to identify oculomotor function (and disturbance) in both healthy and concussed individuals and for a wide variety of potential uses, including as an indicator of overall executive function fatigue, though more testing with the device in varied samples is needed.

Physical fatigue can exert a discernible influence on oculomotor function [11] and manifests as decreased velocity and precision in saccadic eye movements, impacting tasks like pursuit tracking and gaze stabilization [12]. The interplay between fatigue and oculomotor function is further compounded by diminished attention and vigilance, as observed in research on cognitive fatigue [13]. Whether oculomotor tests (particularly those used for detecting concussions) are influenced by physical fatigue remains of interest. Some oculomotor tests, such as the King–Devick test for concussion, show no influence of physical-activity-induced fatigue on test performance results [14], while other tests, like the Vestibular/Ocular Motor Screening (VOMS), do show concussive-like increases in scores after high intensity exercise [11,15]. In addition to the possible identification and recovery management of concussions, EyeGuide claims that their device can identify periods of fatigue and stress [10]. The only published abstract examining high-intensity exercise and EyeGuide Focus performance is by Walshe et al. [16], who did show a significant increase in EyeGuide Focus scores after physical exercise, but only in a small sample (seven athletes) where they completed a 3 min high-intensity warmup protocol. No studies have examined whether this result would also occur after a more real-world sporting scenario, to see whether the EyeGuide Focus may provide a measure for assessing levels of physical and mental processing fatigue. In sports such as rugby union, high levels of physical fatigue and perceived mental stress following a match have been reported previously [17,18], placing it as an ideal sport to test EyeGuide’s claims.

While substantially large deviations in pursuit tracking can occur as a result of concussions, understanding whether a portable, easy-to-use device such as the EyeGuide Focus is capable of detecting smaller disruptions to oculomotor function associated with mental and physical strain is yet to be observed. To understand the typical daily variation of this device’s metric, along with its potential for use outside of concussion research, the intra- and inter-day reliability must first be established, along with the potential influence of engaging in a physically and cognitively demanding activity (i.e., sports competition). Older research shows there is no diurnal variation in oculomotor function [19], although substantial developments in technology suggest the need to confirm this finding. Work by Pearce et al. [20] showed good reliability (ICC = 0.79) of repeat measures within a single session using the EyeGuide Focus, though more recent work by Fuller et al. [21] shows poorer intra-session reliability (ICC = 0.41). Looking at reliability between sessions, Walshe et al. [22] compared EyeGuide Focus performance in two sessions 2–7 days apart, finding quite poor reliability (ICC = 0.24). There is little work examining EyeGuide Focus performance differences across a day, though, which could influence how effective this device is in detecting disruptions to oculomotor function in morning vs afternoon vs evening sporting matches.

As such, the current study has two main aims: (1) to examine the intra-day and inter-day reliability of oculomotor testing using a smooth pursuit task with the EyeGuide Focus device; and (2) to examine the use of EyeGuide Focus pre- and post-rugby union match in female athletes to determine its use as a measurement of overall fatigue. It was hypothesized that the EyeGuide Focus would produce reliable results in Part A of the study, with stable measures across both intra-day and inter-day time points. In Part B, our hypothesis was that the EyeGuide Focus would be sensitive enough to detect fatigue during a game of rugby union, with an increase in EyeGuide Focus scores pre- to post-match.

## 2. Materials and Methods

### 2.1. Participants

In Part A of the study (intra- and inter-day reliability), 20 healthy participants (*M*_age_ = 30 ± 7 years; 11 male, 9 female) volunteered to participate. In Part B of the study, 12 female participants (*M*_age_ = 25 ± 5 years; height 170 ± 6 cm; weight 77 ± 16 kg) completed EyeGuide Focus measurements pre- and post-rugby game. Participants in Part B were classified as trained/developmental athletes and were competing at the local club level [23]. All participants reported normal or corrected-to-normal vision, no medically diagnosed cognitive impairment, and no known concussions in the 3 months prior to participation. Informed written consent was obtained from each participant at the start of their first testing session (for Part A) and before the pre-game EyeGuide Focus testing (for Part B). Institutional ethics approval was obtained for this study (reference numbers HEC21347 and HEC21342).

For Part A, power analyses were conducted. For a within-subjects one-way analysis of variance (ANOVA) with six time points, for a medium effect size (f = 0.25), power of 0.80, and α of 0.05, a sample of 19 participants was needed. For a reliability analysis using intraclass correlation coefficients (ICCs), for an estimated ICC of 0.75 (based on Pearce et al. [14]), power of 0.80, and α of 0.05, a sample of 20 participants was needed. For Part B, the sample size was a practical one based on the number of players involved in the game that was analyzed, so no power analysis was conducted.

### 2.2. Study Design

Part A of this study employed a within-subjects design to assess test–retest reliability of the EyeGuide Focus device, including intra-day, inter-day, and inter-week reliability. Participants completed six identical testing sessions over an 8-day period to assess any changes in EyeGuide Focus performance.

Part B of this study employed a within-subjects cross-sectional design to assess any effects of physical fatigue on EyeGuide Focus performance. Participants completed testing with the EyeGuide Focus before and after a competitive club-level rugby union game.

### 2.3. Eye Tracking Protocol

**Figure 1:**
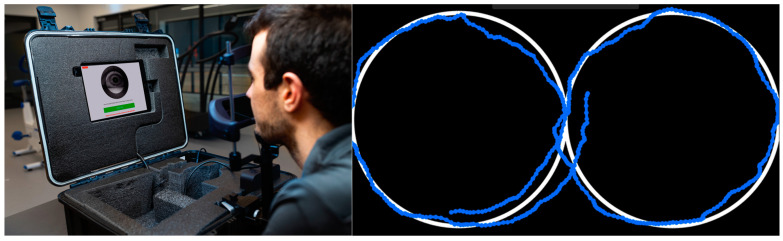
On the left is an example participant set up for EyeGuide Focus protocol, and on the right is an example of participant’s eye-tracking movements (blue) against the stimulus (white).

The eye tracking protocol was performed on a portable, smooth pursuit eye-tracking device known as the EyeGuide Focus (EyeGuide, Lubbock, TX, USA). Participants track a small white dot on a black background moving in an infinity pattern (horizontal figure-8) on an iPad screen for 10 s (Figure 1). Participants completed the task seated comfortably in a dimly lit room, with their face on a chinrest ~50 cm (20 inches) from the iPad screen. Participants were instructed to remain still and not to blink while completing the task. During the task, a fixed camera tracked the eye at 120 Hz, comparing the pupil’s location to the white dot’s location, and gave a score based on how well the dot was tracked. This score was a cumulative distance between the white dot location and pupil location, where a higher score indicated worse tracking (i.e., more error, as the pupil was further away from the dot). After each trial, participants could not see their exact score; however, the software did give a qualitative descriptor of performance (e.g., “Superior”, “High Average”, “Above Average”, “Average”, “Low Average”, “Impaired”, “Severely Impaired”). These descriptors were designed by the manufacturer as groupings based on how far a person’s score was from the average scores of community baseline testing. Three attempts at the task were recorded for each participant at each timepoint (for both Part A and Part B of this study), and the median score was recorded. Trials where eye tracking was lost, or there was a clear blink in the data trace, were deemed unsuccessful, discarded, and repeated.

### 2.4. Procedure

#### 2.4.1. Part A—Reliability

Participants arrived at our laboratory to complete tests at six different sessions across three days. This included two consecutive days (Day 1 and Day 2), and one day a week later (Day 8). Participants completed two sessions per testing day, one in the morning and one in the afternoon. Morning sessions took place between 8am and 10am and afternoon sessions between 2:30 pm and 5 pm. To control for diurnal variations, participants completed the tests at the same time of day for each morning and afternoon session across the three testing days. Each testing session ran for approximately 10 min.

For each morning session, to control for the potential influence of sleep and caffeine consumption, participants first filled out a short survey indicating (1) their quality (rating scale from 1–5) and quantity (approximate hours and minutes) of sleep from the night before and (2) current caffeine consumption (as well as recognizing habitual use). Participants were instructed to try and maintain these variables for all testing days for consistency. After this, participants completed the eye tracking protocol of the EyeGuide Focus outlined in Section 2.3. This was performed in a quiet, dark space. Afternoon sessions followed the same protocol, except only questions about current caffeine consumption were asked, not the questions about sleep. At the end of the sixth session, participants were thanked for their time and debriefed.

#### 2.4.2. Part B—Pre- and Post-Physical Exercise

Part B of this study observed one team competing in a competitive club-level game of rugby union, played in Melbourne, Australia. The game was played at 5 pm in the afternoon in calm weather conditions, and the observed team won the game. All participants wore global positioning system (GPS) devices (Catapult Sports, Vector S7, Melbourne, Australia) throughout the rugby union game. To be included in the analysis, participants were required to play for at least 40 min of the game (the equivalent of one-half of a rugby union game), which excluded one participant. Only active playing time was recorded, with half time, injury time, and substitutions not included in individual players’ game files. To give an indication of the physical player load, GPS data, including total duration, total distance (m), and average speed (m/min), were collected. The majority of participants had not completed EyeGuide Focus testing previously.

First, participants completed a pre-game eye tracking protocol of the EyeGuide Focus outlined in Section 2.3. This was completed immediately before the start of the game and performed in the player’s dressing room with minimal disruptions (i.e., a limited number of people were allowed in the room during testing). Then, participants completed their game of rugby union. Immediately following the game, participants completed post-game EyeGuide Focus testing, which was also completed in the player’s dressing room with minimal disruption. Both EyeGuide Focus sessions (pre and post) were conducted by the same experimenter who checked all tests to ensure accurate test completion (i.e., trace was not lost) but did not record numerical scores. All test scores were downloaded following the completion of the testing. After the post-game testing, participants were thanked for their time and debriefed.

### 2.5. Statistical Analysis

Descriptive statistics are shown as mean ± standard deviation unless stated otherwise. Scores from the eye tracking device are shown as arbitrary units (au). Test–retest typical errors of measurement (TEM) were determined using a Microsoft Excel spreadsheet for reliability [24] and are presented as absolute values along with upper and lower 95% confidence intervals (95%CI). The ICC between trials was also determined in combination with the 95% confidence intervals and interpreted as 0.90 to 1.00 = very high correlation, 0.70 to 0.89 = high correlation, 0.50 to 0.69 = moderate correlation, 0.26 to 0.49 = low correlation, and 0.00 to 0.25 = little, if any correlation [25]. A one-way repeated measures ANOVA with a Bonferroni correction was performed using Jamovi (V2.3, Sydney, Australia) to compare eye tracking performance across all time points for Part A. T-tests were used to compare male vs. female data at each time point for Part A and pre- and post-game for Part B. To observe the influence of match movements on eye tracking performance, a linear regression model was used to observe the change in eye tracking scores from pre- to post-game with covariates for duration, distance, and average speed covered by individual players. Statistical significance for all tests was set at *p* < 0.05. Cohen’s effect sizes (d) were used to describe differences between trials. The magnitude of each effect size was interpreted using thresholds of 0.2, 0.5, and 0.8 for small, moderate, and large, respectively [26]. Where 95% confidence intervals overlapped both small positive and negative effects (±0.2), the effect was deemed unclear.

## 3. Results

### 3.1. Part A—Reliability

**Figure 2:**
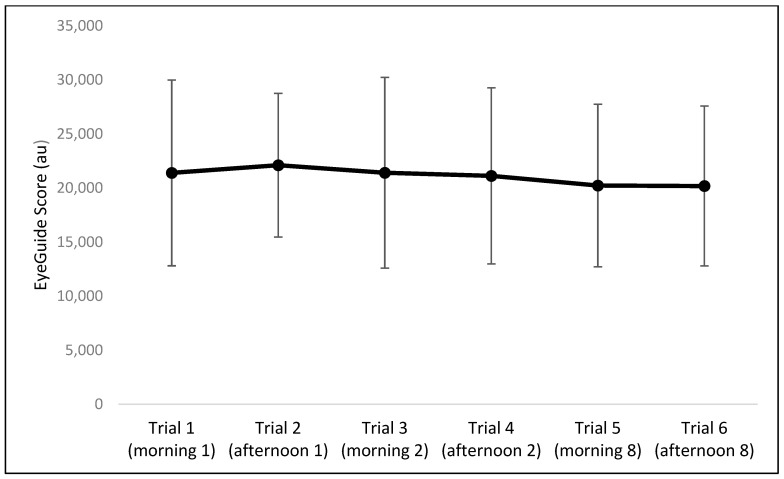
Mean and SD for EyeGuide Focus scores across all six trials. No statistical differences were observed between trials.

The results of the one-way repeated measures ANOVA indicated no significant differences between the six trials for EyeGuide Focus scores (F = 0.09, *p* = 0.99, η^2^ < 0.01; Figure 2) nor any effect for sex (Table 1).

When data were pooled for all morning and afternoon sessions (on Days 1, 2, and 8), there were no significant differences in EyeGuide Focus scores between time points (*p* = 0.92). The intra-day reliability between mornings and afternoons is high (ICC = 0.73), with a TEM of 4098 au (Table 2). The inter-day morning reliability between Day 1 and Day 2 is also high (ICC = 0.79), with a TEM of 4181 au. Inter-day reliability for the afternoon sessions (Day 1 vs. Day 2) resulted in a moderate ICC of 0.58 and a TEM of 4912 au (Table 2). Inter-week reliability (Day 1 vs. Day 8) for morning and afternoon sessions resulted in moderate ICCs (0.59 and 0.66, respectively) and TEMs of 5392 and 4242 au, respectively. The effect size for all comparisons was unclear (Table 2).

### 3.2. Part B—Pre- and Post-Physical Exercise

**Figure 3:**
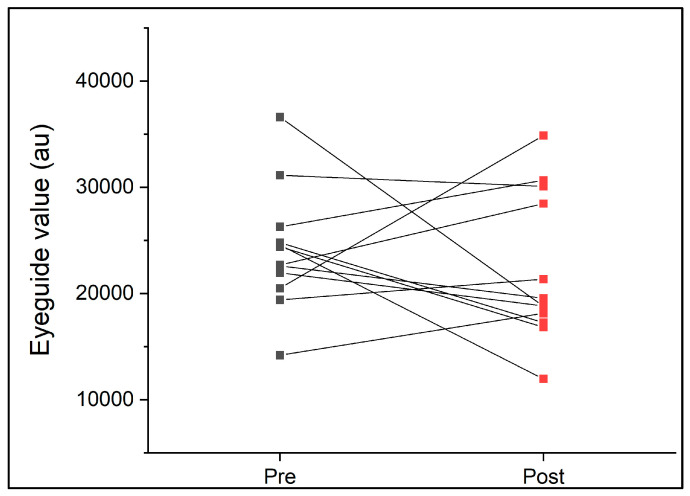
EyeGuide Focus results pre- and post-women’s club-level rugby union game (n = 12).

On average, participants completed 68 ± 12 min of rugby union match play. During the game, participants covered an average of 3841 ± 1038 m (range, 2266–5716 m) at an average speed of 56 ± 10 m.min^−1^ (45–75 m.min^−1^). The EyeGuide Focus values pre- and post-game are presented in Figure 3. There were no significant differences between pre- and post-rugby game eye tracking scores (*p* > 0.05, Table 3) associated with an unclear effect size (d = 0.29 ± 0.41). Further, there was little to no correlation for ICC values pre- to post-game, and the TEM was 6204 au (Table 3). On average, EyeGuide Focus data improved (indicated by lower results) by 1860 au following the game (mean pre 24,091 ± 5670 au; post 22,231 ± 7000 au). GPS variables (total duration, distance, and average speed) did not account for the changes in EyeGuide Focus measures observed (*p* > 0.05, R^2^ = 0.36).

## 4. Discussion

The intra- and inter-day reliability of the EyeGuide Focus appears to be moderate to high, with ICC values between 0.58 and 0.79 and TEM values ranging between 4098 and 5392 au. There were no significant changes in EyeGuide Focus values following fatiguing exercise (a game of rugby union) nor a consistent direction of change observed. There was also no effect of participant sex or the volume or intensity of exercise undertaken (within a reasonably homogenous group) on the EyeGuide Focus results. As such, this paper provides further understanding of the intra- and inter-day reliability of the EyeGuide Focus for how this device can be used for concussion identification. However, fatigue induced as a result of a game of competitive rugby union was not sufficient to be detected using this device, which warrants further research into suitable applications for this technology in examining smaller disruptions to oculomotor functioning due to physical or processing fatigue.

The intra- and inter-day reliability appears to be similar or slightly lower than initially reported intra-test reliability (ICC = 0.79) of the EyeGuide Focus by Pearce et al. [20] and better reliability than more recent studies by Fuller et al. [21] and Walshe et al. [22], who showed ICCs of 0.41 and 0.24, respectively. The differences between these studies may be due to the sample: our study and Pearce et al. [20] used a generally healthy population sample, while Walshe et al. [22] used amateur athletes and Fuller et al. [21] used elite rugby players. It is likely that more athletic samples (particularly those that play contact sports) are exposed to more sub-concussive hits [27,28], which could have an influence on baseline testing (a time point when samples are supposed to be non-concussed) and therefore influence how reliable and consistent baseline scores are. More work examining changes in EyeGuide Focus scores in situations of sub-concussive hits is warranted. Mean test scores were also similar to previous studies using the EyeGuide Focus in adolescent and adult healthy populations (~23,000 au) [20] and slightly better than in adolescent athletes (~30,000 au) [7]. Previous studies assessing other pupillary metrics, including pupil dilation latency, show good reliability within session (~0.91) with sufficient sensitivity to differentiate between immediate stimuli of low, moderate, and high cognitive loads [29]. The greatest reliabilities were shown for intra-day and inter-day morning measures, where there is arguably less potential influence and variation occurring between measures as compared to afternoon measurements or between weeks. The benefits of the EyeGuide Focus, with its moderate-to-high reliability across time of day and between days and weeks, combined with the practical utility of the device being portable and quick (10 s per test), means that there is potential application of this device in a number of areas. However, consideration must be made to the high TEM values (~18–25%), which may limit the ability to observe small changes in this measure as a result of fatigue or otherwise.

It appears that a competitive game of rugby union does not cause any noticeable change in EyeGuide Focus results. Understanding the influence that exercise-related fatigue can have on this measurement is important for the potential application of this device. In the setting of concussion identification, small changes as a result of exercise may not be of concern, as disruptions to the oculomotor system resulting from concussion normally have two-fold or greater changes [7,8]. Other sideline concussion assessment tools that measure oculomotor and cognitive function, such as the King–Devick test and VOMS, have mixed results: for the Kind Devick test, moderate-intensity exercise does not affect test results, and high-intensity exercise actually caused a 5% improvement in test results [30], while for the VOMS, high-intensity exercise increased concussive-like symptoms, though these increases may be primarily driven by levels of reported dizziness [11,15]. The improvement in the King–Devick results is likely due to increased arousal levels following exercise, which can be influenced by both the complexity of the test and the intensity of the exercise [31]. That there are mixed findings in the effect of exercise on other oculomotor concussion tests may provide a reason why there were such mixed responses following the exercise bout in the current study, where 60% of participants improved their results following the game. While there was no relationship with the physical movements performed in-game (playing time, total distance, or speed), we did not capture any physiological measures (e.g., heart rate) to provide an indication of intensity, nor did we capture post-testing data on all players within a specified time period following the cessation of exercise. As such, players who were on the bench prior to the end of the game had more recovery time prior to being tested than those who came straight from the field. Similarly, those who tested first had less recovery time than those who tested at the end, and it is unknown how long the arousing or fatiguing effects of exercise are observed. Further research that observes the influence of controlled-intensity exercise and duration on the EyeGuide Focus results would assist in understanding the utility of this device for application outside of concussion identification and management.

### Limitations and Future Directions

While this study reports on the intra- and inter-day reliability of the EyeGuide Focus, it is important to recognize that this study was completed in a general, healthy population and in sub-elite-level athletes. Both age (adolescence and older populations) as well as competition level may potentially influence the reliability of this device within and between days and may warrant an assessment of reliability within each specific population. Similarly, for Part A, while we tried to capture sleep and caffeine consumption across all days with the intention that this remains consistent for participants, other factors not considered, such as work/personal life stressors or recent exercise, may influence the arousal and cognitive function of participants. While this is part of the typical daily variation that we are measuring, smaller TEMs may be possible if some of these additional factors were controlled for. In Part B, factors like hydration status were also not controlled for, which may have affected results. Finally, EyeGuide Focus performance may have changed over time simply through learning and familiarization with the device. Previous research suggests that familiarization effects may occur more for an adolescent rather than adult sample [20]. We tried to account for familiarization by completing multiple tests at each time point and using the median score of these tests, and the data for Figure 2 suggest that any possible learning effects in Part A were only minor. This is something to keep in mind for future work, however, to ensure sufficient practice trial opportunities are given to participants.

More work examining whether sub-concussive hits can be detected using the EyeGuide Focus is warranted. Controlling for more possible confounding factors on baseline EyeGuide Focus scores, like stress and hydration level, can help us further determine reliability of the device. Determining the best process for how many practice trials to give participants and how many baseline tests (possibly over multiple sessions) are needed to account for any learning or familiarization effects will also help ensure this device is used consistently in practice.

## 5. Conclusions

Overall, this study reports a moderate-to-high reliability of the EyeGuide Focus to observe oculomotor function within-day, between-day, and between-weeks (Part A). As such, there is potential utility of this device for its primary advertised use in game-day concussion detection, particularly partnered with its simplicity, speed, and portability of use. However, there was no difference in EyeGuide Focus scores before and after a rugby match (Part B), suggesting the device may be less effective in detecting more subtle effects on oculomotor function, like fatigue. More research should be conducted to assess the ability of the device to pick up smaller deviations in measurements that may be the result of physical or cognitive fatigue.

## Figures and Tables

**Table 1:** Mean (±SD) EyeGuide Focus scores at each of the six testing timepoints, for all participants and then separated by female and male participants, with *p*-value for sex comparisons.

	All Participants	Female (n = 9)	Male (n = 11)	*p*-Value
Morning Day 1	21,398 ± 8594	20,176 ± 8516	22,498 ± 8967	0.57
Afternoon Day 1	22,115 ± 6648	20,580 ± 4352	23,372 ± 8060	0.36
Morning Day 2	21,413 ± 8818	17,400 ± 5705	25,024 ± 9798	0.06
Afternoon Day 2	21,125 ± 8144	18,871 ± 5158	22,969 ± 9819	0.27
Morning Day 8	20,235 ± 7523	16,677 ± 5536	23,438 ± 7870	0.06
Afternoon Day 8	20,188 ± 7392	19,092 ± 5992	21,084 ± 8553	0.57
Overall Mean	20,751 ± 7657	18,799 ± 5895	23,064 ± 8844	-

**Table 2:** Mean intraclass correlation coefficients (ICCs), typical error of measurement (TEM) as absolute values, and effects sizes for each comparison (intra-day, inter-day, and inter-week reliability for morning and afternoon testing sessions).

Reliability Measure	ICC	TEM (au)	Effect Size (Cohen’s d)
Intra-day (mornings vs. afternoons)	0.73 (0.58–0.83)	4098 (3469–5007)	−0.06 (−0.37–0.35)
Inter-day morning (Day 1 vs. Day 2)	0.79 (0.53–0.91)	4181 (3159–6183)	0.00 (−0.64–0.64)
Inter-day afternoon (Day 1 vs. Day 2)	0.58 (0.20–0.81)	4912 (3741–7184)	0.13 (−0.58–0.66)
Inter-week morning (Day 1 vs. Day 8)	0.59 (0.20–0.82)	5392 (4074–7974)	0.16 (−0.59–0.69)
Inter-week afternoon (Day 1 vs. Day 8)	0.66 (0.31–0.85)	4242 (3226–6196)	0.27 (−0.54–0.71)

Note. All data shown are mean values, with 95% confidence intervals in brackets.

**Table 3:** Pre- and post-rugby union EyeGuide Focus scores, with mean intraclass correlation coefficients (ICCs), typical error of measurement (TEM) as absolute values, and an effect size for the pre- to post-game comparison.

Pre EyeGuide Focus Score (AU)	Post EyeGuide Focus Score (AU)	ICC	TEM (au)	*p*-Value	Effect Size (Cohen’s d)
24,091 ± 5670	22,231 ± 7000	0.06 (−0.54–0.61)	6204 (4395–10535)	0.48	0.29 (−0.69–0.92)

Note. Pre- and post-EyeGuide Focus scores are shown as mean ± SD. ICC, TEM, and Cohen’s d data are mean values, with 95% confidence intervals in brackets.

## Data Availability

Data for this study are available upon reasonable request.

## Abbreviations

The following abbreviations are used in this manuscript:

ICC	Intraclass correlation coefficient
M	Mean
SD	Standard deviation
GPS	Global positioning system
TEM	Typical error of measurement
CI	Confidence interval
ANOVA	Analysis of variance
